# Coronavirus or Cholangitis? An Acute Necrotizing Encephalopathy Caused by COVID-19

**DOI:** 10.7759/cureus.44448

**Published:** 2023-08-31

**Authors:** Ryotaro Watanabe, Junki Mizumoto, Akira Ohya

**Affiliations:** 1 Internal Medicine, Mimihara General Hospital, Sakai, JPN; 2 Family Medicine, International Research Center for Medical Education, Graduate School of Medicine, The University of Tokyo, Tokyo, JPN; 3 General Practice, Mimihara General Hospital, Sakai, JPN

**Keywords:** infectious disease, covid-19, cognitive bias, clinical reasoning, cholangitis, acute necrotizing encephalopathy

## Abstract

A 63-year-old Japanese woman presented to the emergency room with a fever and altered mental status. She was diagnosed as acute cholangitis and coronavirus disease 2019 (COVID-19). On the second day, her consciousness level deteriorated. The patient was finally diagnosed with acute necrotizing encephalopathy (ANE). This case illustrated ANE caused by COVID-19 that co-occurred with acute cholangitis. ANE is a subtype of acute encephalitis/encephalopathy, sometimes related to COVID-19. ANE shares some clinical features with acute cholangitis. COVID-19 and bacterial infections may coexist, thus complicating an accurate diagnosis. Physicians should avoid overlooking life-threatening febrile conditions even if the diagnosis of COVID-19 is confirmed.

## Introduction

Acute necrotizing encephalopathy (ANE) is an acute encephalopathy/encephalitis usually developed after viral infection. Influenza is the most common cause of ANE, followed by HHV-6 infection. It is most frequently reported in children [1]. However, many cases of ANE due to the coronavirus disease 2019 (COVID-19) have been reported in adults, and it is one of the fatal complications of COVID-19 [2-3]. Pulmonary complications are a well-known complication of coronary infection, but neurologic complications are also important.

## Case presentation

**Figure 1:**
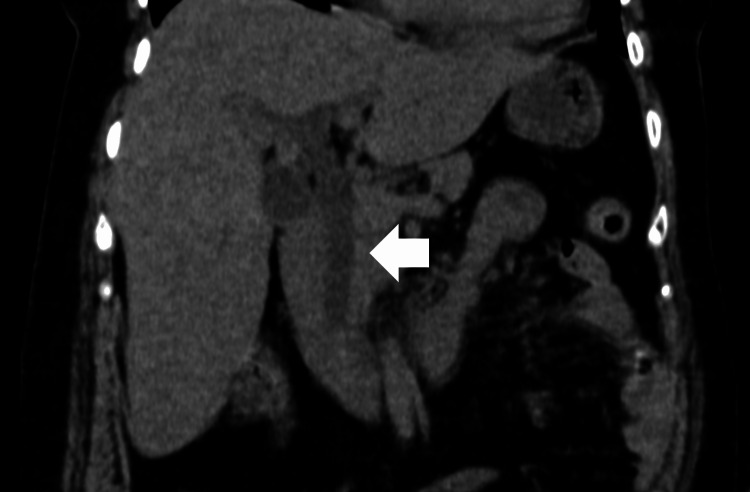
Computed tomography (CT) of the abdominal on admission demonstrates dilatation of the common bile duct (white arrow).

**Figure 2:**
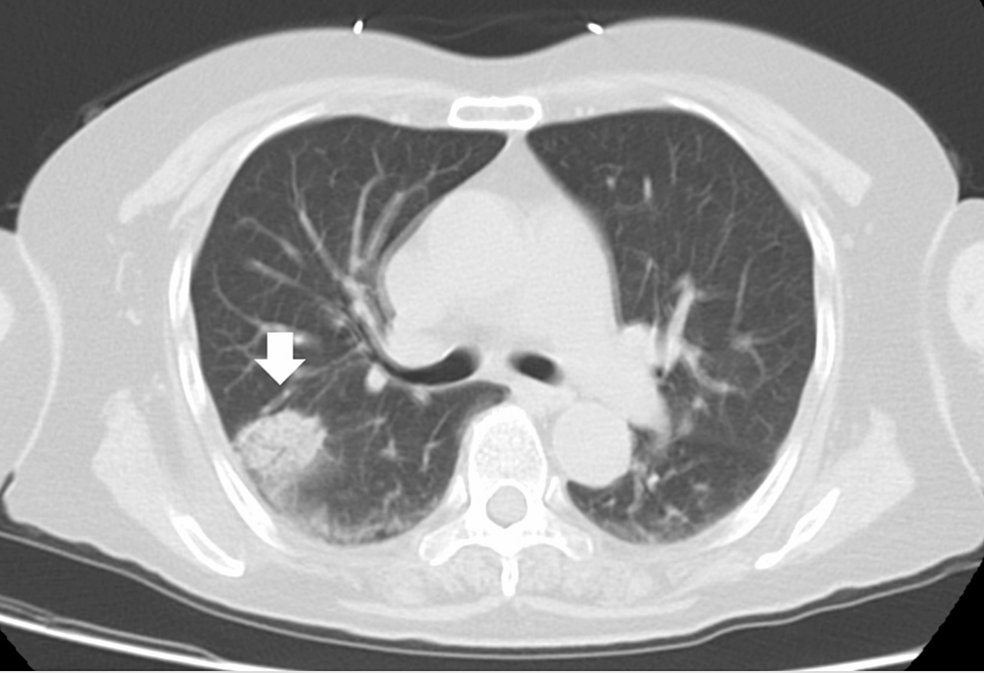
CT scan showed consolidation in the upper lobe of the right lung (white arrow).

A 63-year-old Japanese woman presented to the emergency room with a fever and altered mental status (AMS) that had persisted since that morning. She showed no cough, increased sputum production, dyspnea, or abdominal pain. The patient had a history of hypertension and was using calcium channel blockers and diuretics regularly. On initial evaluation, she appeared sick and somnolent and could not pronounce her name or date. She had no tenderness in the abdomen and no abnormal breath sounds. The Glasgow Coma Scale (GCS) was 14 (E4V4M6). Her vital signs were body temperature, 38.8℃; blood pressure, 128/70 mmHg; heart rate, 110 beats per minute; respiratory rate, 24 beats per minute; and oxygen saturation, 98%, while breathing 2 L/min of oxygen. Physical examination revealed no signs of meningeal irritation or neurological abnormalities except AMS. Laboratory tests showed an elevation in the levels of hepatobiliary enzymes (Table 1). The result of venous blood gas analysis was: pH was 7.488; pCO_2_ was 42.9 mmHg; pO_2_ was 21.9 mmHg; pHCO_3_ was32.5 mmHg and Lactate was 1.2 mmol/L (reference range: 0.44- 1.78 mmol/L). Computed tomography (CT) showed choledocholithiasis with dilatation of the common bile duct (Figure 1) and consolidation in the upper lobe of the right lung (Figure 2).

**Table 1:** Laboratory results

Basic Labs	Results	Reference Range
White cell count (WBC)	10700 /μL	4000-8000 /μL
C-reactive protein level (CRP)	1.38 mg/dL	0.00-0.30 mg/dL
Aspartate aminotransferase (AST)	402 IU/L	9-35 IU/L
Alanine aminotransferase (ALT)	210 IU/L	5-33 IU/L
Lactate dehydrogenase (LDH)	502 IU/L	124-222 IU/L
Total Bilirubin (T-Bil)	1.2 mg/dl	0.2-1.2 mg/dl
Alkaline phosphatase (ALP)	2434 IU/L	100-340 IU/L
γ-glutamyl transpeptidase (γ-GTP)	41 IU/L	0-30 IU/L
Prothrombin time (PT)	11.3 second	10.0-13.0 second
Activated partial thromboplastin time (APTT)	30.6 second	25-40 second

**Figure 3:**
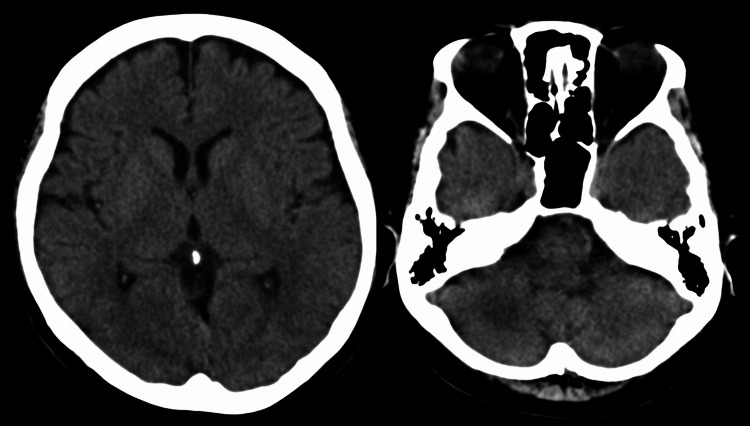
Computed tomography (CT) of the brain on admission.

In contrast, CT of the head showed no abnormalities (Figure 3). Emergency endoscopic retrograde cholangiopancreatography revealed a 5 mm gallstone in the common bile duct, and only Stenting was performed. Blood culture showed the growth of Escherichia coli. Therefore, a tentative diagnosis of sepsis due to acute cholangitis was made. Considering the COVID-19 epidemic, a SARS-CoV-2 antigen quantification test was performed, which was positive. Subsequently, meropenem and remdesivir were administered, and the patient was hospitalized.

**Figure 4:**
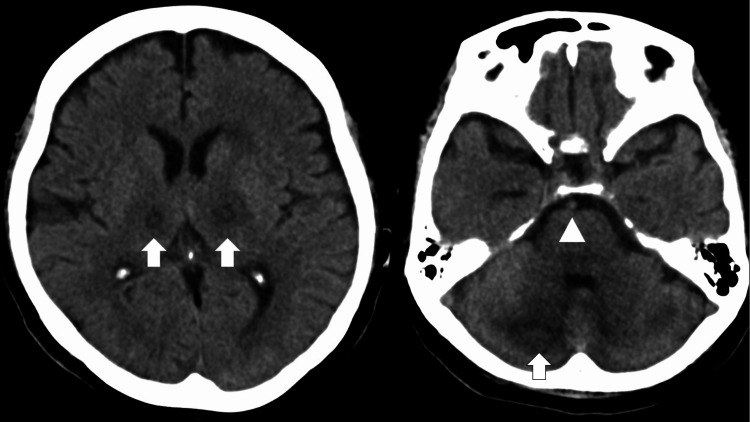
Brain CT on the second day. Low-dense areas in the bilateral thalamus and the cerebellum (white arrow) and a hyperdense right middle cerebral artery sign (white arrowhead) were seen.

**Figure 5:**
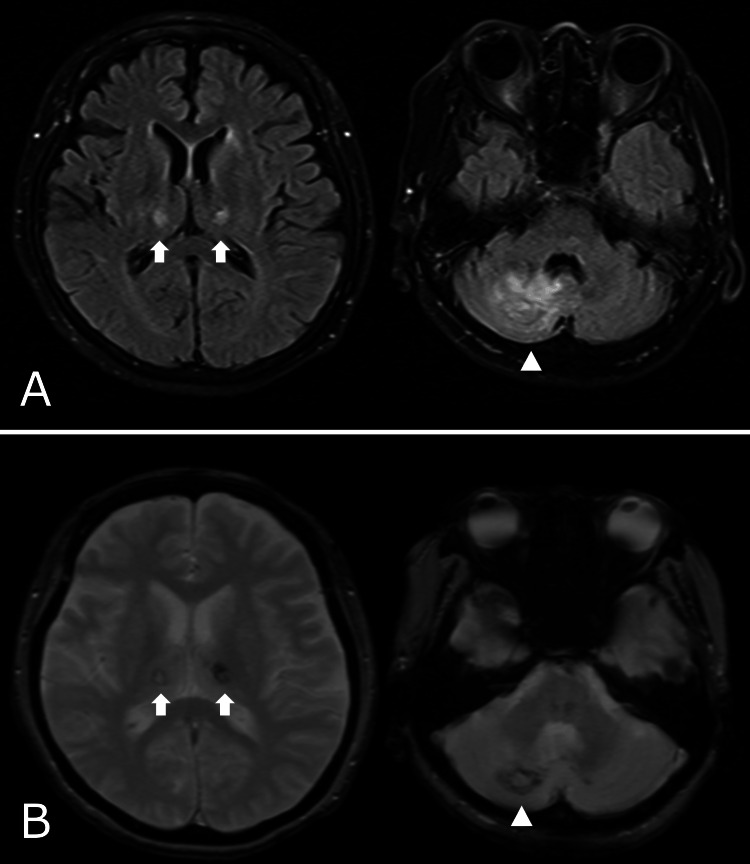
Brain MRI on the day 28. (A) Fluid attenuated inversion recovery imaging showed highly dense areas in the right cerebellar hemisphere (white arrowhead) and bilateral thalamus (white arrow). (B) T2-weighted short-tau inversion recovery imaging showed heterogeneous low-dense areas in the same regions (white arrow and white arrowhead).

On the second day post-admission, her consciousness level deteriorated to GCS 10 (E2V2M6). Physical examination revealed a newly developed nuchal rigidity. A cerebrospinal fluid test revealed a cell count of 1 /μl, protein at 114 mg/dl, and glucose at 76 mg/dl. Head CT showed low-density areas in the bilateral thalamus and cerebellum and focal hyperdense of the middle cerebral artery (MCA) (Figure 4). Therefore, acute encephalitis/encephalopathy and top of the basilar syndrome were suspected. Methylprednisolone (mPSL) 1000 mg/day, immunoglobulin 400 mg/kg/day, and heparin 0.5 ml/h were administrated. Subsequently, the patient began to have respiratory failure and was incubated. ThemPSL dose was reduced to 60 mg/day on day 7 and changed to dexamethasone 6.6 mg/day on day 8 to treat COVID-19 pneumonia. To prevent adrenal failure, the dexamethasone dose was reduced to 3.3 mg/day on day 10 and discontinued on day 12. The patient's consciousness improved to GCS 10 (E4VTM6) on day 15, and she was extubated on day 22. Magnetic resonance imaging (MRI) of the head on day 28 showed highly dense areas in the right cerebellar hemisphere and bilateral thalamus on fluid-attenuated inversion recovery imaging (Figure 5A) and heterogeneous low-density areas in the same region on T2-weighted short-tau inversion recovery imaging (Figure 5B). These findings led to a diagnosis of ANE. On day 31, the patient was transferred to the rehabilitation ward because of improvement in her general condition. The patient recovered and could live a daily life after intensive rehabilitation, although her short-term memory and attention remained slightly impaired. The patient was discharged on day 90 of hospitalization.

## Discussion

This case illustrated ANE caused by COVID-19, whose clinical features, laboratory data, and neuroimages fulfilled the diagnostic criteria of ANE [4]. Other differential diagnoses, such as septic emboli and acute disseminated encephalomyelitis (ADEM), were not likely. Septic cerebral embolism is often associated with infective endocarditis and often presents with multiple cerebral infarctions. However, in the present case, there was no evidence of verrucous lesions on echocardiography, and the intracranial lesions were symmetrically distributed, so septic cerebral embolism was ruled out [5]. Brain MRI in ADEM causes asymmetric high-signal lesions on T2-weighted images, but this case is not considered ADEM because of the symmetric lesion [4].

ANE is a subtype of acute encephalitis/encephalopathy [6] and occasionally develops post-COVID-19 [2-3]. Imaging tests play an important role in diagnosing ANE: bilaterally symmetric thalamic lesions are essential and highly characteristic of ANE. During the acute period, thalamic lesions have (i) low densities on CT, (ii) low intensities on T1-weighted images, and high intensities on T2-weighted MRI [7-8]. These findings are reflected in pathological necrosis with perivascular hemorrhage in the central area, edema, and tissue roughening in the surrounding area [7].

COVID-19 and bacterial infections may coexist, thus complicating an accurate diagnosis [9]. Clinical symptoms and signs of ANE in the early stages include fever, AMS, and elevated AST, ALT, and LDH levels. These enzymes are elevated in 85% of patients who developed ANE and are clinically significant in some cases [10]. Since these features are common with acute cholangitis, diagnosing ANE during the early stages was difficult in this patient.

Early diagnosis and therapeutic intervention in ANE, including administration of steroids within 24 hours of onset, may lead to better patient outcomes [11]. In this case, prolonged AMS, despite appropriate cholangitis treatment, was crucial for reconsidering a tentative diagnosis. Overcoming diagnostic pitfalls, including confirmation bias and premature disclosure, helps clinicians make a prompt and correct diagnosis [12].

## Conclusions

 We reported an ANE resulting from COVID-19 that accidentally co-occurred with acute cholangitis. ANE and acute cholangitis have certain common features, including fever, AMS, and elevated liver enzymes. Physicians should recognize that, in the COVID-19 era, an accurate diagnosis of ANE may be complicated by bacterial infection. Therefore, ANE should be suspected and investigated in patients with COVID-19 who develop prolonged or progressive AMS despite appropriate treatment.

## References

[1] Hoshino A, Saitoh M, Oka A (2012). Epidemiology of acute encephalopathy in Japan, with emphasis on the association of viruses and syndromes. Brain Dev.

[2] Mullaguri N, Sivakumar S, Battineni A, Anand S, Vanderwerf J (2021). COVID-19 related acute hemorrhagic necrotizing encephalitis: a report of two cases and literature review. Cureus.

[3] Agha T, Moon JY, Iyer N (2022). Acute necrotizing encephalopathy secondary to COVID-19. Proc (Bayl Univ Med Cent).

[4] Mizuguchi M, Ichiyama T, Imataka G (2021). Guidelines for the diagnosis and treatment of acute encephalopathy in childhood. Brain Dev.

[5] Elsaghir H, Khalili Y (2023). Septic Emboli.

[6] Mizuguchi M, Abe J, Mikkaichi K, Noma S, Yoshida K, Yamanaka T, Kamoshita S (1995). Acute necrotising encephalopathy of childhood: a new syndrome presenting with multifocal, symmetric brain lesions. J Neurol Neurosurg Psychiatry.

[7] Mizuguchi M, Hayashi M, Nakano I (2002). Concentric structure of thalamic lesions in acute necrotizing encephalopathy. Neuroradiology.

[8] Albayram S, Bilgi Z, Selcuk H (2004). Diffusion-weighted MR imaging findings of acute necrotizing encephalopathy. AJNR Am J Neuroradiol.

[9] Brown L (2020). COVID Blindness. Diagnosis (Berl).

[10] Mizuguchi M (1997). Acute necrotizing encephalopathy of childhood: a novel form of acute encephalopathy prevalent in Japan and Taiwan. Brain Dev.

[11] Okumura A, Mizuguchi M, Kidokoro H (2009). Outcome of acute necrotizing encephalopathy in relation to treatment with corticosteroids and gammaglobulin. Brain Dev.

[12] Mangus CW, Mahajan P (2022). Decision making: Healthy heuristics and betraying biases. Crit Care Clin.

